# An ecological model in support of an ontology of mental functioning

**DOI:** 10.1371/journal.pmen.0000407

**Published:** 2026-01-16

**Authors:** Maryanne J. Sacco, Guy Divita, Kathleen Coale, Howard H. Goldman, Carolyn P. Rosé

**Affiliations:** 1 Rehabilitation Medicine Department, Clinical Center, National Institutes of Health, Bethesda, Maryland, United States of America; 2 Department of Psychiatry, University of Maryland, School of Medicine, Baltimore, Maryland, United States of America; 3 Language Technologies Institute and Human-Computer Interaction Institute, School of Computer Science, Carnegie Mellon University, Pittsburgh, Pennsylvania, United States of America; PLOS: Public Library of Science, UNITED KINGDOM OF GREAT BRITAIN AND NORTHERN IRELAND

## Abstract

Health records contain rich sources of mental health data that can be used to evaluate disability and health care outcomes. However, a lack of behavioral health ontologies focused on daily life activity functioning has impeded development of clinical informatic tools to extract mental functioning information. We aim to present the theoretical foundation and conceptual model upon which the Ecological Mental Functioning Ontology (EMFO) was built to facilitate natural language processing (NLP) to extract mental functioning information in free-text clinical records. Subject matter experts operationally defined mental functioning, and a related theoretical perspective was established. Face validity of a proposed model was obtained using an iterative grounded theory approach. An annotation schema based on the model was constructed and tested using manual annotation and consensus on datasets of real and synthetic clinical notes. An annotation schema, based on the Ecological Model of Mental Functioning (EMMF), was shown to be robust when using NLP methods to identify and extract mental functioning information in real and synthetic behavioral health clinical notes. Mental functioning is a complex phenomenon that is fully conceptualized within an ecological milieu encompassing the dynamic transactive relationship between the person, the nature and demands of activities the person participates in, and the external contextual and environmental factors within which the activities take place. By operationalizing mental functioning, the EMMF provided a conceptual roadmap to develop the EMFO and NLP methods that identify and extract mental functioning activity information in clinical records.

## Introduction

The impact that mental health disorders have on the life and living of the person, and the lives of those around them and society, is immense. Mental health conditions contribute to poorer health outcomes, premature death, human rights violations, and global and national economic loss [1,2]. Recently, due to the Covid pandemic, rates of common conditions such as depression and anxiety increased by more than 25%, adding to the nearly one billion people worldwide who were already living with a mental disorder [3]. Globally however, mental health care lags behind other aspects of health care that targets illnesses associated with physical functioning. These disparities are due to several factors including discrimination and stigma associated with mental illness [4] and fewer resources worldwide allocated for mental health care [5].

Mental functioning is a critical component of mental and behavioral health and is reflected in one’s functioning and behaviors in daily life activities. The World Health Organization (WHO) recognizes functioning at the activities and participation level as a key central outcome of health [6], and it is at this level of functioning that our paper will focus.

Accessing mental health data on individual and population levels is necessary if health outcomes are to be evaluated and informed by science. A rich source of mental health data exists in mental health records. Natural language processing (NLP) methods can be employed to identify and extract mental health-related mentions found in clinical text and turn them into countable categorized and standardized structured data. Beyond identifying the important text to focus on, the categorized counted mentions found by NLP techniques provide aggregatable data across electronic health records (EHR), corpora for tasks involving clinical support, research, epidemiology, policy, compliance and quality assurance. NLP methods often rely on particular domain ontologies that provide knowledge and a common understanding of the phenomenon of interest, concepts and their relationships. Mental functioning ontologies facilitate NLP methods by standardizing annotations and guiding the data extraction of mental functioning information in free-text clinical records.

A review of current ontologies in behavioral health and sciences, however, found no mental functioning ontology whose primary focus aligns with mental functioning at the activities and participation level as defined in the International Classification of Functioning, Disability and Health (ICF) [6]. Though ontologies related to behavioral science have been developed, such as emotions [7], cognition [8], and neuroscience [9], their domains of interest are targeted at ICF classifications of body functions, body structures or health conditions. Other than the upper-level category of “behavior” in the Mental Functioning Ontology [10], of which “behavior” has not been further classified, there is no mental functioning ontology whose primary focus aligns with the ICF activities and participation component. Nor is there a mental functioning ontology that classifies mental functioning from a more holistic and ecological perspective that broadens activity functioning within particular environments and contexts.

In their consensus study report on the state of ontologies in the behavioral sciences in the domain of mental health, the National Academies of Sciences [11] found that there are far fewer ontologies in the behavioral health domain than any other scientific domains. The committee notes that “progress in the behavioral sciences has been hindered by the use of different terms or descriptions for the same underlying entity or condition; the use of the same term for different entities or concepts; the use of different, poorly correlated measures for the same entity; and the use of measures whose relationship to the phenomena they are measuring is not well understood” [11, p. 2].

To support clinical NLP (cNLP) efforts to extract mental functioning information at the activities and participation level in clinical records, we have developed and distributed the Ecological Mental Functioning Ontology (EMFO) [12] that classifies mental functioning concepts and their relationships to one another. In this paper we describe the Ecological Model of Mental Functioning (EMMF) that details the underlying theoretical perspective and conceptual framework upon which the Ecological Mental Functioning Ontology (EMFO) was built.

The applicability of the EMMF will be demonstrated through the lens of annotations done in synthetic health records with a use case of disability determination for social security work benefits. The EMMF is equally applicable to a range of other outcomes such as vocational rehabilitation where a broader perspective and broader model would be useful.

## Objectives

The aims of this paper are to: (1) define mental functioning and differentiate key concepts and their relationships for ontology building; (2) present the theoretical foundation and conceptual model that was developed as a framework for building the Ecological Mental Functioning Ontology; and (3) provide a conceptualization of mental functioning that could structure the development of clinical informatic evidence-review tools for disability determination.

## Methods

### Defining mental functioning

A Mental Health Working Group (MHWG) was formed internally in the Epidemiology and Biostatistics Section of the Rehabilitation Medicine Department (RMD) at the National Institutes of Health (NIH) Clinical Center and tasked with defining and operationalizing the concept of mental functioning at the activities level. The group represented behavioral health, rehabilitation, medicine, computer science, and health research professions. Mental functioning was defined as the “*mind-directed observable external behaviors that represent what people actually do (or do not do) in response to decisions occurring during real-time transactions as they unfold moment by moment in daily life and over time*” [13, p. 1].

### Establishing a theoretical perspective

The MHWG reviewed, explored, and discussed the applicability of several existing frameworks, theories, and models to conceptualize mental functioning at the activities level. Sources for the exploratory review included the International Classification of Functioning, Disability and Health (ICF) [6], person-environment-activities transactive models [14–16], social-ecological theories [17,18], and Open System Theory [19]. A lack of classification of personal factors and differentiation of context, environments, activities, and participation concepts in the ICF proved challenging for conceptualizing mental functioning from a whole-person perspective [13]. Due to the wide variety of mental functioning stakeholder backgrounds and disciplines, no one theoretical base from any particular discipline or taxonomy was sufficient to provide a shared ontological understanding of mental functioning from perspectives as varied as psychopharmacology, social psychology, biomedicine, and behavioral and ecological-environmental sciences.

To foster a shared understanding of mental functioning, the General Systems Theory of Open Systems [19] was selected by the MHWG as the primary framework due to its broader applicability and relevance to a wide variety of mental functioning stakeholders from different backgrounds, disciplines, and traditions. This model provided a scaffold or framework upon which other theories, perspectives, taxonomies, and terminologies could be draped to further complement and expand the understanding, conceptualization, and study of mental functioning at the activities level. The Open System model provides a conceptualization of functioning as a circular process with 4 major constructs: Input, Throughput, Output and Feedback [19]. Open Systems Theory stipulates that an organism, or human in this case, is shaped by one’s environment and in turn also affects one’s environment in a continuous loop of human-environment transaction.

As one of the most internationally recognized classification taxonomies to describe and define functioning, disability and health, the ICF was adopted to further our conceptualization of activities and participation functioning as a distinct type of functioning from other types of physiologic functions that occur at anatomical levels of body structures and body functions. We aligned all ICF functioning concepts within the Open Systems model and developed a first draft of the Ecological Model of Mental Functioning for describing mental health functioning.

### Establishing face validity

We established face validity of the EMMF using the grounded theory method [20] iterative process of presenting the model in professional forums to audiences made up of diverse and relevant stakeholders (i.e., subject matter experts, clinicians, health care researchers and computer scientists), obtaining feedback, and using feedback to revise the model. We used clinical queries cased in real life or case scenarios, such as “Why does someone overreact or act defensively during interpersonal interactions that most others would not perceive as hostile?”, to test mental functioning concepts and their relationships. This process was repeated until no further feedback requiring refinement was obtained and audiences were satisfied with the current model and deemed it credible.

The validation process began with audiences from internal and inward-facing contexts (the MHWG, and clinicians in the Rehabilitation Medicine Department at the Clinical Center of the NIH) to more public, external, and outward facing contexts encompassing multi-disciplinary perspectives and included: occupational therapists at a professional state conference; disability adjudicators and policy-makers at the U.S. SSA; and multi-disciplinary members of the Mental Health Medical Informatics working group of the American Medical Clinical Informatics Association representing behavioral health and computer sciences.

### Annotation process

An EMMF annotation schema was developed and tested by human coders on a subset of 10 real clinical notes from the NIH Clinical Center. These notes were purposely sampled from a previously manually annotated dataset for the most annotation mentions in the Self-care and Domestic Life ICF domains. This domain was selected as it most represented activity performance in everyday life. An EMMF annotation guideline was developed alongside to facilitate consistent annotation decisions toward a reliability effort. An iterative process was used to achieve annotation consensus among the first and third authors, who had clinical expertise. This process involved independent manual annotations by the two authors on the same 10 clinical note dataset. The annotations were compared for discrepancies and discussed among the human annotators to reach consensus. This resulted in a gold standard corpus of the 10 real clinical notes that contains the consensus dataset. The second author, a computer scientist, contributed to consensus discussions, guideline development, and helped to refine the model and the EMFO.

## Results

This work has resulted in the development of a proposed Ecological Model of Mental Functioning (EMMF) that was applied to extract concepts of, and related to, mental functioning in clinical documentation using NLP annotation methods. This framework emerged from the need to link evidence of mental functioning to observed behavior in daily life activities, for purposes such as disability determination; and for the need to highlight vital concepts, such as Person factors and Feedback that are essential to fully comprehend the idiosyncratic nature of mental functioning and the person’s ability to affect change and adapt.

The EMMF has four key assumptions: (1) functioning at the whole-person level of participation in activities involves an intricate interplay between a person’s mental and physical functioning abilities; (2) mental functioning is recognized as a complex and dynamic process with an important temporal component; (3) the EMMF appreciates that what is adaptive and maladaptive behavior is dependent upon whose perspective is being adopted, i.e., the person’s “insider” perspective, or the “outsider” perspective of others in the social environment; (4) mental functioning as “mind-driven” behaviors refer to actions a person takes that are within mental control and consciousness. We exclude behaviors that are reflexive or automatic.

**Fig 1:**
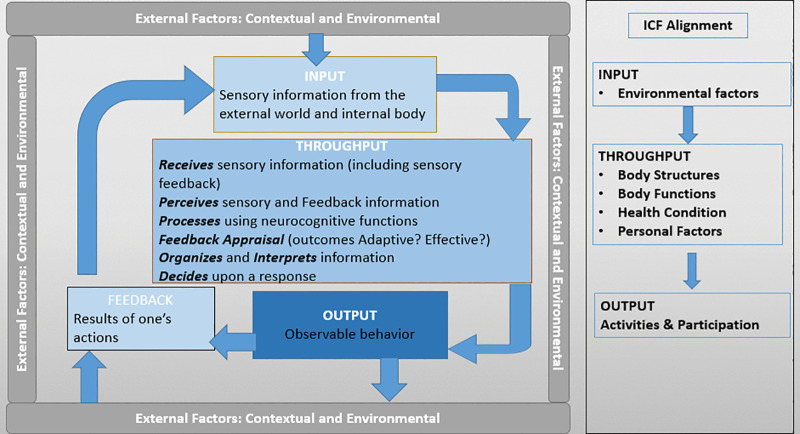
The Ecological Model of Mental Functioning and alignment with the ICF.

The EMMF adopts a hierarchical structure of concepts and categories which are External Factors, Input, Throughput, Output and Feedback. This becomes important for the development of the EMFO and future work that will further classify concepts within this hierarchical structure. **Fig 1** illustrates the EMMF with higher-level concepts and their relationship, including a side bar of how all ICF components of function were rearranged and mapped for conceptual coherence and clarity.

### Applying the ecological model of mental functioning

The usability of the EMMF for natural language processing is applied using a fictional case of a Navy veteran, Mr. David Williams, diagnosed with post-traumatic stress disorder (PTSD) and mild traumatic brain injury (mTBI). He is married with children, and he is having difficulties reintegrating into a civilian work environment. For the purposes of this paper, two synthetic notes from occupational therapy and psychiatry were generated for the case by domain experts and with assistance using ChatGPT [21]. A case example was drafted by the first author that included person and background factors and context such as the age, service area, job type and the dual diagnoses known to often have sequala of mental functioning impacting cognitive and interpersonal skills. This was inputted into ChatGPT using cues to develop the case including multiple deployments and traumatic experiences leading to PTSD, and multiple blast injuries leading to mTBI with resulting symptoms. This cue was repeated 3 times to obtain case variations. Selected parts of each ChatGPT variation were used to provide the traumatic event, and signs and symptoms sequala. The case was augmented by the first author, resulting in the following case scenario:

*David Williams is a 36-year-old Navy veteran who served two tours in a combat zone as a Medic. He was exposed to multiple blasts over the course of his deployments and began experiencing symptoms such as headaches, light sensitivity, difficulty concentrating and dizziness consistent with mild TBI. Shortly before his last tour ended, the Humvee that was travelling only a few hundred meters in front of his was hit by an IED and exploded, killing all five occupants. He and his unit were tasked with cleaning the wreckage and collecting the remains of his dead comrades. Shortly after this he began experiencing symptoms of PTSD including flashbacks and nightmares, hypervigilance, and insomnia. He completed his tour and was sent to the National Intrepid Center of Excellence at Walter Reed National Military Medical Center for further assessment and treatment of his PTSD and TBI. He eventually received an honorable discharge and was recently employed as a health technician at a VA medical center.*

Using this case, ChatGPT was cued to write an occupational therapy assessment note and a psychiatric encounter note with additional cues such as being married with two children ages 8 and 10, having difficulties in family life and work as a health technician due to his symptoms. These cues were repeated a few times to generate variations, each time tweaking the cues to generate further desired details. Using selected parts of the different variations, an occupational therapy assessment and psychiatry encounter note were drafted and augmented by the first author. The notes were reviewed by domain experts and finalized.

**Fig 2:**
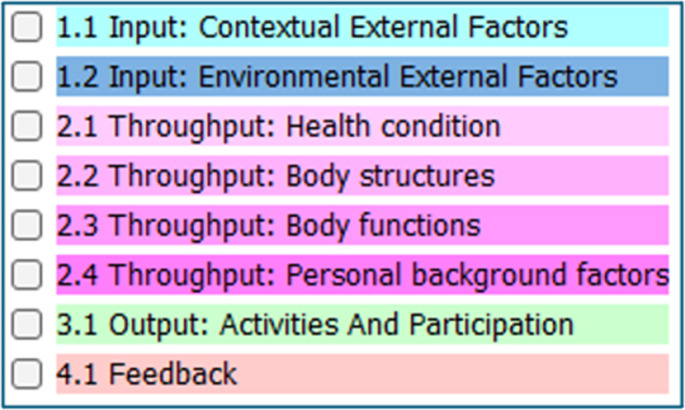
EMMF annotation schema.

These synthetic notes were manually annotated by the clinical annotator authors using the EMMF annotation schema incorporating entity concepts of Input, Throughput, Output and Feedback, and their aligned ICF concepts. **Fig 2** of the EMMF annotation schema below shows the EMMF annotation schema and the color legend for annotations. The GATE software tool was used for annotation [22].

In annotation, the least span of text to represent a concept is ideal. The surrounding text provides contextual information and data extraction of a word or two can be viewed within the context of the note. Separate entity annotations can overlap, and this accounts for the color changes seen in the note.

**Fig 3:**
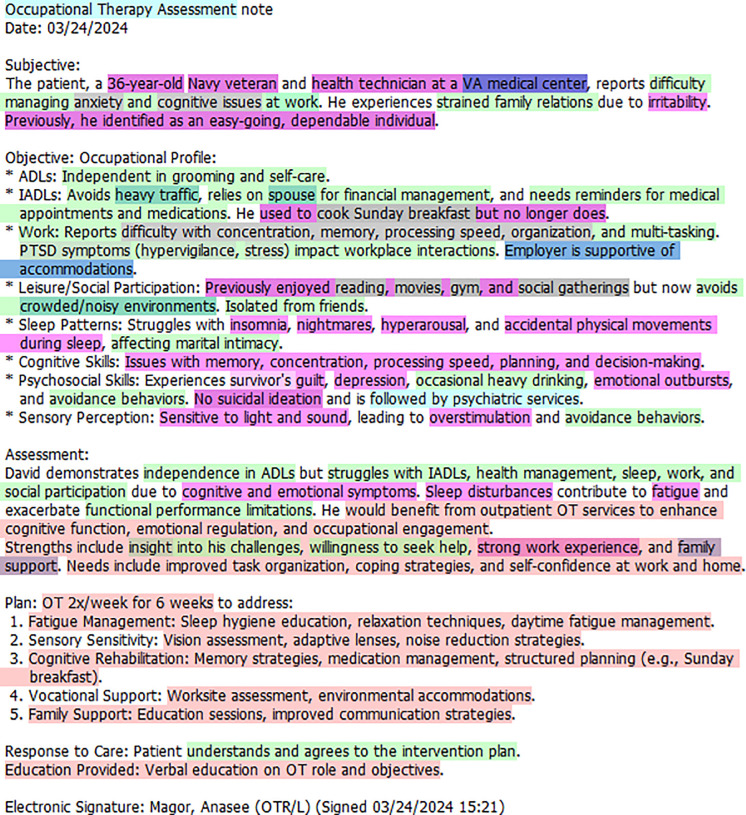
Annotated occupational therapy assessment note.

**Fig 4:**
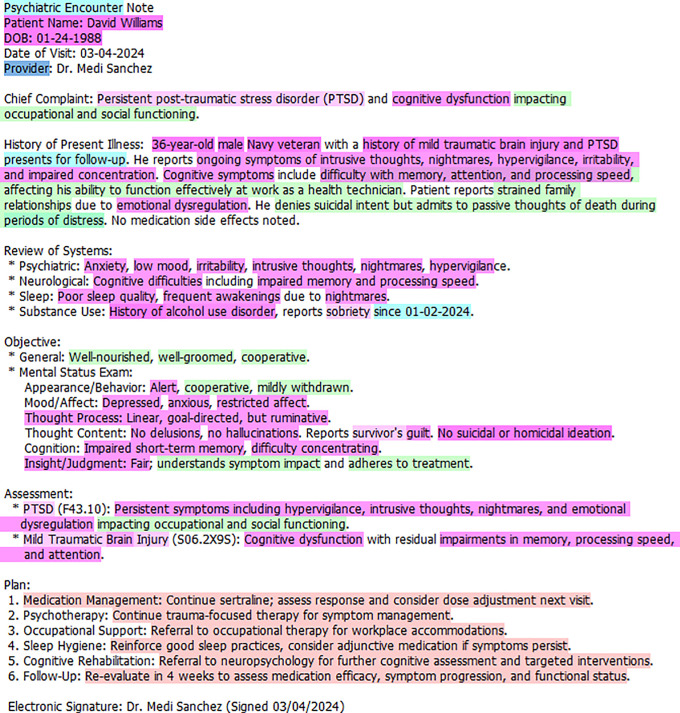
Annotated psychiatric encounter note.

In the following section the EMMF is applied using the synthetically developed occupational therapy assessment note (see **Fig 3**) and a psychiatric encounter note (see **Fig 4**) that have been annotated using the EMMF schema. For the purposes of this paper, the notes were condensed to one-page with the assistance of ChatGPT. Please refer to **Fig 2**, the EMMF schema, that provides the color legend representing annotated concepts. As the open system model is circular, we will begin by describing Output where mental functioning is observed.

### Output

Mental functioning as Output is operationalized in the EMMF as the outward observable behaviors of the activities one does and the life areas one participates in (ICF activities and participation). Annotations of activity functioning have for the most part followed annotation guidelines in free-text health records for coding the ICF activity domains for Mobility [23], Self-care and Domestic Life [24], Interpersonal Interactions and Relationships [25], and Communication and Cognition [26] representing the three combined ICF domains of Learning and Applying Knowledge, General Tasks and Demands, and Communication. Quantitative metric details for inter-rater reliability can be found in each annotation guideline. Clinical NLP performance metrics of precision, recall, and F1 scores can be found as reports on two corpora in the EpiBio GitHub repository [12]: the cNLP report on a sample of work disability claimants [27]; and a cNLP report on the Gold Standard Corpus for activity information (GoSCAI) [28]. **Table 1** below illustrates clinical text examples annotated in the occupational therapy and psychiatry notes for Output: Activities and Participation and the 9 ICF activities and participation domains they represent.

**Table 1:** ICF Activity domains with clinical text representation.

ICF Code	Output: ICF Activities and Participation domains	Represented annotated text in occupational therapy and/or psychiatric note
d1	Learning and applying knowledge	*Insight into his challenges* [OT] *Understands symptom impact* [Psych]
d2	General tasks and demands	*Avoids heavy traffic* [OT] *…reports difficulty with…multitasking* [OT]
d3	Communication	*Understands and agrees to the intervention plan* [OT] *…admits to passive* (suicidal) *thoughts* [Psych]
d4	Mobility	none
d5	Self-care	*Independent in grooming and self-care* [OT] *Well-nourished* [Psych]
d6	Domestic life	*Needs reminders for medical appointments and medications* [OT] *Struggles with IADLs* [OT]
d7	Interpersonal interactions and relationships	*Isolated from friends* [OT] *Strained family relationships* [Psych]
d8	Major life areas	*Relies on spouse for financial management* [OT] *Difficulty with memory, attention and processing speed affecting his ability to function effectively at work as a health technician* [Psych]
d9	Community, social and civic life	none

### Input

One can appreciate that mental functioning does not occur in a vacuum. External factors impose demands and exert influence upon the person that can support or hinder performance. External contextual and environmental factors provide Input as sensory information the person directly receives through the body from one’s immediate and proximal social and physical environments and contextual situation (ICF environmental factors). Displayed conceptually as the outer border in the EMMF, external factors represent the outer world in which one lives, functions, interacts, and participates in life activities.

Contextual factors were operationalized for annotation as a reference to a particular place, at a particular time, in a particular situation, including the situation of the present moment the note was written. Physical environments are tangible features in the environment and can be as varied as references to a setting such work or to tangible objects such as bills, traffic, etc. Social environments are other people in the person’s life and social relationships/roles. **Table 2** below illustrates clinical text examples annotated in the occupational therapy and psychiatry notes for Input: Contextual or Environmental external factors.

**Table 2:** Input External Factors with clinical text representation.

Input: External Factors	Represented annotated text in occupational therapy and/or psychiatric note
Input: Contextual External Factors	*Occupational therapy assessment* [OT] *Presents for follow up* [Psych]
Input: Environmental External Factors (physical)	*VA medical center* [OT] *Heavy traffic* [OT]
Input: Environmental External Factors (social)	*Employer is supportive of accommodations* [OT] *Provider* [Psych]

### Feedback

Feedback is a special kind of Input derived specifically from Outputs (actions or non-actions) and provides information about the performance of Outputs. Feedback is tied to real-time information about the result of one’s actions or inactions (Output) within a certain situational context, environment, and point in time. Feedback from one’s external and internal world provides continual information about the results of one’s actions and behavior, allowing the person to self-regulate and adjust their behaviors and actions in real time. There is no concept for Feedback in the ICF.

External feedback was operationalized for annotation as what the provider (or others) would recommend based on an assessment to address the patient’s problems or needs. Usually found in recommendations, interventions, or prognosis sections, Feedback information can include what the provider has done or plans to do with the assumption that this information was communicated to the patient. Only the section of text that is feedback of an action of what the patient should do, participate in etc., is annotated. Text referring to actions of the provider, such as “refer the patient for” would be excluded. Text was considered External Feedback if an imaginary suffix such as “I recommend that…” or “In my opinion, you/the patient needs…” could be placed before the annotated text.

Internal feedback was operationalized as sensations stemming from the body that occur as a result of actions, thoughts, or what is occurring in the environment, such as feeling pain after a fall, feeling upset after receiving bad news, or feeling fear when awakened by a loud unexpected noise. In the EMMF annotation schema we did not separate external and internal feedback, and there were no annotations for internal feedback in the synthetic notes presented. For conceptual purposes we provide clinical text examples in **Table 3** below, to show how internal feedback might be represented. To further clarify External Feedback conceptually, an imaginary prefix (in parentheses) is inserted in the annotated text example.

**Table 3:** Feedback with clinical text representation.

Feedback	Represented annotated text in occupational therapy and/or psychiatric note
External Feedback	*(you) would benefit from outpatient OT services to enhance cognitive function, emotional regulation and, occupational engagement* [OT] *(you need) Medication management: Continue sertraline; assess response and consider dose adjustment next visit* [Psych]
Internal Feedback	*She feels better when she takes her medications* *Just thinking about talking with her mother makes her very anxious* He feels irritable when having to communicate with his ex-wife

### Throughput

One’s body is the gateway to experiencing the world, both externally and internally. The external world constitutes everything outside of one’s body. The internal world constitutes the body itself, everything from the skin inwards. Throughput is conceptualized as the processes within one’s body where external and internal sensory information is received in the anatomical brain (ICF body structure) as Input and perceived, processed, and organized using mental psychological functions (ICF body functions). Every individual is unique in their physical and mental condition (ICF health condition), life experiences, situation, and psychological makeup (ICF personal factors) and this has a salient bearing in how sensory information is interpreted, with the mind integrating all functions and factors, and deciding how to act upon that information.

In the EMMF, we consider ICF classifications of body structures and functions, health condition and personal factors to be intrinsic to each individual and therefore we categorize them as Person Factors and adopt the ICF classification and definitions for these concepts. However, the ICF lacks a formal classification of personal factors. We therefore conceptualize Personal Background Factors as a contextual factor that is unique to the individual and includes demographic, biographical, and psychological factors. Demographic factors may include age, gender, race, developmental stage, or living situation. Biographical factors pertain to one’s life story and history, personal life experiences, social roles, education, personal culture, etc. Psychological factors have to do with one’s locus of control, interests, will, intent, volition, behavioral patterns, habits, and routines, beliefs and values and meaning-making. Feedback appraisal, as part of Throughput, are the internal mental functions of judgement and evaluation of one’s actions. Feedback appraisal uses internal and external Feedback, that is sensory Input, to determine (i.e., appraise) how adaptive or maladaptive one’s actions were to the situation and would be operationalized in annotation as mentions of body functions pertaining to judgement and insight.

In **Table 4** below, we provide Throughput concepts in the EMMF schema and operational definitions for annotation, with added clinical text examples taken from the annotated OT and psychiatry notes.

**Table 4:** Throughput with operational definitions and clinical text representation.

Throughput	Operational definition	Annotated text example
Health Condition	Includes acute or chronic disease, disorder, injury or trauma and other relevant information such as signs and symptoms. Includes health states, such as pregnancy, sobriety, aging, stress, congenital anomaly, or genetic predisposition.	Cognitive and emotional symptoms [OT] PTSD [Psych] Sobriety [Psych]
Body Structures	Anatomical parts of the body such as organs, limbs, and their components.	Brain [Psych]
Body Functions	Global mental functions include orientation, intellectual functions, psychosocial functions, energy and drive functions, personality and temperament functions, and sleep functions. Specific mental functions include attention, memory, psychomotor functions, emotional and perceptual functions, thought functions, mental functions of language, calculation functions and higher-level executive cognitive functions.	Irritability [OT] Issues with memory, concentration, processing speed, planning and decision-making [OT] Emotional outbursts [OT] Ongoing symptoms of intrusive thoughts, nightmares, hyperviligence, irritability and impaired concentration [Psych] Restricted affect [Psych] Poor sleep quality [Psych] Cognitive dysfunction [Psych]
Personal Background - demographic	Demographic factors are annotated as separate variables such as age, social status (married), role, (veteran) family status (with children).	36-year-old [OT/Psych] Navy veteran [OT/Psych] Health technician at the VA medical center [OT]
Personal Background - psychological	Psychological factors that are unique to the individual such feelings, beliefs, values, likes and dislikes, etc.	Survivors guilt [OT]
Personal Background - biographical	A person’s history or story that is past but continues to influence current mental functioning. A past context, a previous life experience not part of the current context.	Previously identified as an easy-going dependable individual [OT] Strong work experience [OT] History of alcohol use disorder [Psych]

Personal background factors can also provide a context that is unique to the individual. This is often documented in the patient history notes but can be found in other sections pertaining to client factors that provide a story, life experience, or background that provide insight into the person’s past experiences.

We operationalize personal biographical factors to include a person’s history or story that is past but continues to influence current mental functioning. For example, mentions in the note pertaining to the traumatic events experienced is annotated. These mentions describe a past context, a previous life experience. As they are not part of the current context, they are annotated as a past personal background factor.

Demographic factors are annotated as separate variables such as age (36-year-old), social status (married), role, (veteran) family status (with children). Psychological factors annotated include strong feelings of survivor’s guilt and themes of recurring thought content.

**Fig 5:**
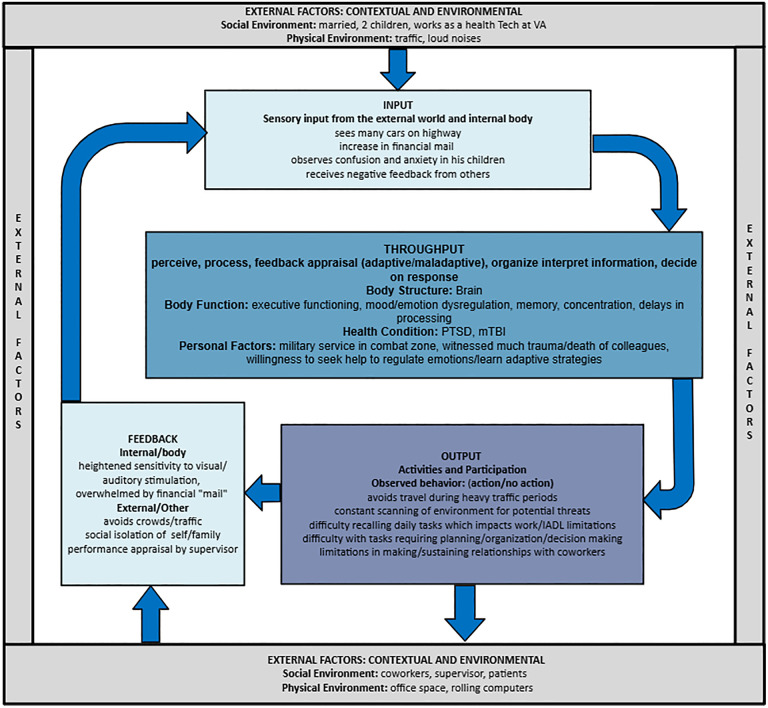
EMMF and case example.

In **Fig 5** below we have provided a more comprehensive picture of the case example with additional functional information placed in the EMMF. The ICF concepts have been integrated into the model, and richer examples of the full case study to illustrate the concepts have been inserted.

## Discussion

Functioning in everyday life activities can only be fully understood when examined within the ecological milieu that underpins the transactive dynamic relationship between the person, the nature and type of activities the person engages in, and the environment and context within which the activities are taking place. In the EMMF, we have shown that though mental functioning occurs at the observed behavioral level (Output), it can only be fully understood if all other aspects of person-environment-activities interaction that are taking place are considered (Input, Throughput, and Feedback). This has significant implications for determining work disability for persons with mental health disorders, cognitive or intellectual disabilities. Mental functioning is completely grasped when considering the effects of external factors, such as the social and physical environments, or person factors that support or hinder participation in life activities, such as work.

The EMMF was developed as the theoretical foundation to lay the groundwork and establish the Ecological Mental Functioning Ontology (EMFO) [29] which was developed and published to support NLP efforts to extract mental functioning information at the activities and participation level in large volume datasets of health records. Ranallo and Tenenbaum [30] highlighted the significant gaps in extraction systems that capture mental health knowledge and concept representation and provide a roadmap for enhancing system technologies that provide computation representation of mental health knowledge and data. The EMMF provides a conceptualization of mental functioning that is broad enough to be understood by a diverse set of mental health stakeholders and addresses some of the gaps outlined by Ranallo and Tenenbaum.

Our proposed EMMF should be viewed in the context of the work of other investigators advancing ontological work in behavioral science. Dr. Hastings and colleagues [31,32] have been instrumental in highlighting the need for the development of mental functioning ontologies to address the many challenges of data management and data-driven research using computer-assisted science. Toward these efforts, a Mental Functioning Ontology (MF) and Mental Disease Ontology (MD) were developed to describe human mental functioning and disease and place mental functioning as a central concept, within a broader biomedical scientific context. The MF is an upper-level ontology that aims to represent all aspects of mental functioning including behavior, mental processes, and mental anatomical structures, with cognition, perception and emotion actively being developed. The MD ontology incorporates bio-chemical-mechanical aspects of mental health, and the related concepts within terminology platforms (i.e., SNOMED and DSM), which primarily codify the pathologies involved with mental functioning.

Ontologies in behavioral health have primarily focused on domains that would be classified as body structures and functions, and health conditions, within the World Health Organization’s (WHO) classification of functioning, disability, and health (ICF) [6]. The same can be said for clinical NLP that has used terminology platforms such as SNOMED [33] and UMLS [34] with efforts primarily designed for the extraction and curation of data that can assist clinical decision-making for diagnosis, prediction, and therapeutic drug interventions. Though these perspectives and efforts are commendable and of great importance, the focus has not been on whole-person functioning that occurs in daily life activities.

Efforts toward a mental functioning ontology has application beyond health informatics, NLP, and clinical care. A shared vocabulary has implications for scientific discourse and the synthesis and retrieval of valuable developing knowledge. Without ontological clarity, research findings may be difficult to compare, replicate and generalize. Clinical natural language processing (cNLP), guided by a theoretical model and an established ontology, can be instrumental in these efforts by automating the identification, extraction, and aggregation of a particular phenomenon of interest, such as mental functioning information, in large volume datasets.

## Limitations

Limitations included a small dataset of 10 real clinical records and two synthetic clinical notes that the testing and annotations using the EMMF schema were based upon. Though consensus of EMMF annotations were achieved among three coauthors, we did not establish statistical inter-rater reliability (IRR) measures. Future work to further test the EMMF schema and establish IRR measures would strengthen the EMMF schema and annotation guidelines.

## Conclusions

Mental functioning is an important aspect of behavioral health and a critical indicator of one’s functioning in daily life activities. Mental functioning information at the activity level exists in electronic health records as data that can be extracted using NLP methods and analyzed by researchers for purposes such as disability determination and to improve behavioral health outcomes for persons and populations.

To support NLP methods to identify and extract mental functioning information at the activity level in health records, a domain ontology is needed that is grounded in a theoretical perspective. We have presented The Ecological Model of Mental Functioning (EMMF), developed as the conceptual foundation upon which Ecological Mental Functioning Ontology was based that can be used in NLP to identify and extract activity and mental functioning information.

## Supporting information

S1 Table Visual alternative for Figure 3: Annotated occupational therapy assessment note. (DOCX)

S2 Table Visual alternative for Figure 4: Annotated Psychiatric encounter note. (DOCX)
