# Supplementary material for: An ecological model in support of an ontology of mental functioning
Source: PLOS Ment Health. 2026 Jan 16;3(1):e0000407. doi: 10.1371/journal.pmen.0000407 (PMC12810788; doi:10.1371/journal.pmen.0000407)
Supplement: S1 Table — (DOCX) [file pmen.0000407.s001.docx]

# S1 Table

## Visual alternative to Figure 3: Annotated occupational therapy assessment note.

This 2-column table provides a visual alternative to Figure 3 by omitting color highlights of coded annotation entities assigned to clinical text. The occupational therapy assessment note of Figure 3 is shown in a two-column table. The first column is the text selected for annotation, the second column indicates whether annotations were assigned to the text, and if so, which schema entities were assigned. As text can be assigned more than one entity, the text that overlaps in two entities is repeated separately.

| **Free-text in OT note** | **Coded Annotation entities** |
| --- | --- |
| Occupational Therapy Assessment | Input: Contextual external factors |
| Note. Date: 03/24/2024. Subjective: The patient, a | not annotated |
| 36-year old | Throughput: Personal background factors |
| Navy veteran | Throughput: Personal background factors |
| and | Not annotated |
| Health technician at a VA medical center | Throughput: Personal background factors |
| VA medical center | Input: Environmental external factors |
| reports | Not annotated |
| difficulty managing anxiety and cognitive issues at work | Output: Activities and participation |
| anxiety | Throughput: Body function |
| cognitive issues | Throughput: Body function |
| at work | Input: Contextual external factors |
| He experiences | Not annotated |
| strained family relations | Output: Activities and participation |
| due to | Not annotated |
| irritability | Throughput: Body Function |
| Previously, he identified as an easy-going, dependable individual | Throughput: Personal background factors |
| Objective: Occupational Profile: ADLs: | Not annotated |
| Independent in grooming and self-care | Output: Activities and participation |
| IADLs | Not annotated |
| Avoids heavy traffic | Output: Activities and participation |
| Heavy traffic | Input: Environmental external factors |
| relies on spouse for financial management | Output: Activities and participation |
| spouse | Input: Environmental external factors |
| and | Not annotated |
| needs reminders for medical appointments and medications | Output: Activities and participation |
| He | Not annotated |
| used to cook Sunday breakfast but no longer does | Throughput: Personal background factors |
| cook Sunday breakfast | Output: Activities and participation |
| Work: Reports difficulty with concentration, memory, processing speed, organization, and multi-tasking | Output: Activities and participation |
| difficulty with concentration, memory, processing speed, organization | Throughput: Body Function |
| PTSD symptoms (hypervigilance, stress) impact workplace interactions | Output: Activities and participation |
| PTSD symptoms | Throughput: Health condition |
| hypervigilance | Throughput: Health condition |
| stress | Throughput: Health condition |
| Employer is supportive of accommodations | Input: Environmental external factors |
| Leisure/Social Participation | Not annotated |
| Previously enjoyed reading, movies, gym, and social gatherings | Throughput: Personal background factors |
| reading | Output: Activities and participation |
| movies | Output: Activities and participation |
| gym | Output: Activities and participation |
| Social gatherings | Output: Activities and participation |
| But now | Not annotated |
| avoids crowded/noisy environments | Output: Activities and participation |
| crowded/noisy environments | Input: Environmental external factors |
| Isolated from friends | Output: Activities and participation |
| Sleep Patterns: Struggles with | Not annotated |
| insomnia | Throughput: Body function |
| nightmares | Throughput: Body function |
| hyperarousal | Throughput: Body function |
| and | Not annotated |
| accidental physical movements during sleep | Throughput: Body function |
| affecting marital intimacy | Output: Activities and participation |
| Cognitive Skills | Not annotated |
| Issues with memory, concentration, processing speed, planning, and decision-making | Throughput: Body function |
| Psychosocial Skills Experiences | Not annotated |
| survivor’s guilt | Throughput: Health condition |
| guilt | Throughput: Body function |
| depression | Throughput: Body function |
| depression | Throughput: Health condition |
| occasional heavy drinking | Output: Activities and participation |
| emotional outbursts | Throughput: Body function |
| and | Not annotated |
| avoidance behaviors | Output: Activities and participation |
| No suicidal ideation | No suicidal ideation |
| and is | Not annotated |
| followed by psychiatric services | Input: Contextual external factors |
| Sensory Perception | Not annotated |
| Sensitive to light and sound | Throughput: Body function |
| leading to | Not annotated |
| overstimulation | Throughput: Body function |
| and | Not annotated |
| avoidance behaviors | Output: Activities and participation |
| Assessment: David demonstrates | Not annotated |
| independence in ADLs | Output: Activities and participation |
| but | Not annotated |
| struggles with IADLs, health management, sleep, work, and social participation | Output: Activities and participation |
| Due to | Not annotated |
| cognitive and emotional symptoms | Throughput: Body function |
| Sleep disturbances | Throughput: Body function |
| contribute to | Not annotated |
| fatigue | Throughput: Body function |
| and exacerbate | Not annotated |
| functional performance limitations | Output: Activities and participation |
| He | Not annotated |
| would benefit from outpatient OT services to enhance cognitive function, emotional regulation, and occupational engagement | Feedback |
| Strengths include insight into his challenges, willingness to seek help, strong work experience, and family support | Feedback |
| insight into his challenges | Output: Activities and participation |
| insight | Throughput: Body function |
| willingness to seek help | Output: Activities and participation |
| strong work experience | Throughput: Personal background factors |
| family support | Input: Environmental external factors |
| Needs include improved task organization, coping strategies, and self-confidence at work and home | Feedback |
| Plan | Not annotated |
| OT 2x/week for 6 weeks | Feedback |
| to address | Not annotated |
| 1: | Not annotated |
| Fatigue Management: Sleep hygiene education, relaxation techniques, daytime fatigue management | Feedback |
| 2. Sensory Sensitivity: | Not annotated |
| Vision assessment, adaptive lenses, noise reduction strategies | Feedback |
| 3. | Not annotated |
| Cognitive Rehabilitation: Memory strategies, medication management, structured planning (e.g., Sunday breakfast) | Feedback |
| 4. Vocational Support: | Not annotated |
| Worksite assessment, environmental accommodations | Feedback |
| 5. | Not annotated |
| Family Support: Education sessions, improved communication strategies | Feedback |
| Response to Care: Patient | Not annotated |
| understands and agrees to the intervention plan | Output: Activities and participation |
| Education Provided: Verbal education on OT role and objectives | Feedback |
| Electronic Signature: Magor, Anasee (OTR/L) (Signed 03/24/2024 15:21) | Not annotated |
