# Supplementary material for: An ecological model in support of an ontology of mental functioning
Source: PLOS Ment Health. 2026 Jan 16;3(1):e0000407. doi: 10.1371/journal.pmen.0000407 (PMC12810788; doi:10.1371/journal.pmen.0000407)
Supplement: S2 Table — (DOCX) [file pmen.0000407.s002.docx]

# S2 Table

## Visual alternative to Figure 4: Annotated psychiatric encounter note.

This 2-column table provides a visual alternative to Figure 4 by omitting color highlights of coded annotation entities assigned to clinical text. The psychiatric encounter note of Figure 4 is shown in a two-column table. The first column is the text selected for annotation, the second column indicates whether annotations were assigned to the text, and if so, which schema entities were assigned. As text can be assigned more than one entity, the text that overlaps in two entities is repeated separately.

| **Free-text in Psychiatric Encounter note** | **Coded Annotation entities** |
| --- | --- |
| Psychiatric Encounter | Input: Contextual external factors |
| Note | Not annotated |
| Patient Name: David Williams | Throughput: Personal background factors |
| DOB: 01-24-1988 | Throughput: Personal background factors |
| Date of Visit: 03-04-2024 | Not annotated |
| Provider | Input: Environmental external factors |
| Dr. Medi Sanchez | Not annotated |
| Chief Complaint | Not annotated |
| Persistent post-traumatic stress disorder (PTSD) | Throughput: Health condition |
| and | Not annotated |
| cognitive dysfunction | Throughput: Health condition |
| impacting occupational and social functioning | Output: Activities and participation |
| History of Present Illness | Not annotated |
| 36-year-old | Throughput: Personal background factors |
| male | Throughput: Personal background factors |
| Navy veteran | Throughput: Personal background factors |
| with a | Not annotated |
| history of mild traumatic brain injury and PTSD | Throughput: Personal background factors |
| mild traumatic brain injury | Throughput: Health condition |
| brain | Throughput: Body structure |
| PTSD | Throughput: Health condition |
| presents for follow-up | Input: Contextual external factors |
| He reports | Not annotated |
| ongoing symptoms of intrusive thoughts, nightmares, hypervigilance, irritability, and impaired concentration | Throughput: Body functions |
| Cognitive symptoms | Throughput: Health condition |
| include | Not annotated |
| difficulty with memory, attention, and processing speed, affecting his ability to function effectively at work as a health technician | Output: Activities and participation |
| difficulty with memory, attention, and processing speed | Throughput: Body functions |
| Patient reports | Not annotated |
| strained family relationships | Output: Activities and participation |
| due to | Not annotated |
| emotional dysregulation | Throughput: Body functions |
| He | Not annotated |
| denies suicidal intent but admits to passive thoughts of death during periods of distress | Output: Activities and participation |
| periods of distress | Input: Contextual external factors |
| No medication side effects noted | Not annotated |
| Review of Systems: Psychiatric: | Not annotated |
| Anxiety | Throughput: Body functions |
| low mood | Throughput: Body functions |
| Irritability | Throughput: Body functions |
| intrusive thoughts | Throughput: Body functions |
| Nightmares | Throughput: Body functions |
| hypervigilance | Throughput: Body functions |
| Neurological: | Not annotated |
| Cognitive difficulties | Throughput: Body functions |
| including | Not annotated |
| impaired memory and processing speed | Throughput: Body functions |
| Sleep: | Not annotated |
| Poor sleep quality | Throughput: Body functions |
| frequent awakenings | Throughput: Body function |
| due to | Not annotated |
| nightmares | Throughput: Body function |
| Substance Use: | Not annotated |
| History of alcohol use disorder | Throughput: Personal background factors |
| alcohol use disorder | Throughput: Health condition |
| reports | Not annotated |
| sobriety | Throughput: Health condition |
| since 01-02-2024. | Input: Contextual external factors |
| Objective: General: | Not annotated |
| Well-nourished | Output: Activities and participation |
| well-groomed | Output: Activities and participation |
| cooperative | Output: Activities and participation |
| Mental Status Exam: Appearance/Behavior: | Not annotated |
| Alert | Throughput: Body function |
| Cooperative | Output: Activities and participation |
| mildly withdrawn | Output: Activities and participation |
| Mood/Affect: | Not annotated |
| Depressed | Throughput: Body function |
| anxious | Throughput: Body function |
| restricted affect | Throughput: Body function |
| Thought Process: Linear, goal-directed, but ruminative | Throughput: Body function |
| Thought Content: | Not annotated |
| No delusions | Throughput: Body function |
| no hallucinations | Throughput: Body function |
| Reports | Not annotated |
| survivor's guilt | Throughput: Health condition |
| guilt | Throughput: Body function |
| No suicidal or homicidal ideation | Not annotated |
| Cognition: | Not annotated |
| Impaired short-term memory | Throughput: Body function |
| difficulty concentrating | Throughput: Body function |
| Insight/Judgment: Fair | Throughput: Body function |
| understands symptom impact | Output: Activities and participation |
| and | Not annotated |
| adheres to treatment | Output: Activities and participation |
| Assessment: | Not annotated |
| PTSD | Throughput: Health condition |
| (F43.10): | Not annotated |
| Persistent symptoms including hypervigilance, intrusive thoughts, nightmares, and emotional dysregulation | Output: Activities and participation |
| impacting occupational and social functioning | Throughput: Body function |
| Mild Traumatic Brain Injury | Throughput: Health condition |
| Brain | Throughput: Body structure |
| (S06.2X9S): | Not annotated |
| Cognitive dysfunction | Throughput: Health condition |
| with residual | Not annotated |
| impairments in memory, processing speed, and attention | Throughput: Body function |
| Plan: | Not annotated |
| 1. | Not annotated |
| Medication Management: Continue sertraline; assess response and consider dose adjustment next visit | Feedback |
| 2. Psychotherapy: | Not annotated |
| Continue trauma-focused therapy for symptom management | Feedback |
| 3. Occupational Support: | Not annotated |
| Referral to occupational therapy for workplace accommodations | Feedback |
| 4. Sleep Hygiene: | Not annotated |
| Reinforce good sleep practices, consider adjunctive medication if symptoms persist | Feedback |
| 5. Cognitive Rehabilitation: | Not annotated |
| Referral to neuropsychology for further cognitive assessment and targeted interventions | Feedback |
| 6. Follow-Up: | Not annotated |
| Re-evaluate in 4 weeks to assess medication efficacy, symptom progression, and functional status | Feedback |
| Electronic Signature: Dr. Medi Sanchez (Signed 03/04/2024) | Not annotated |
